# Impact of anion exchange adsorbents on regional citrate anticoagulation

**DOI:** 10.1177/0391398820947733

**Published:** 2020-08-13

**Authors:** Karin Strobl, Stephan Harm, Ute Fichtinger, Claudia Schildböck, Jens Hartmann

**Affiliations:** Center for Biomedical Technology, Department for Biomedical Research, Danube University Krems, Krems, Austria

**Keywords:** Adsorption, anion exchange resin, artificial liver support, blood purification, calcium, regional citrate anticoagulation

## Abstract

**Introduction:**

Heparin and citrate are commonly used anticoagulants in membrane/adsorption based extracorporeal liver support systems. However, anion exchange resins employed for the removal of negatively charged target molecules including bilirubin may also deplete these anticoagulants due to their negative charge. The aim of this study was to evaluate the adsorption of citrate by anion exchange resins and the impact on extracorporeal Ca^2+^ concentrations.

**Methods:**

Liver support treatments were simulated in vitro. Citrate and Ca^2+^ concentrations were measured pre and post albumin filter as well as pre and post adsorbents. In addition, batch experiments were performed to quantify citrate adsorption.

**Results:**

Pre albumin filter target Ca^2+^ concentrations were reached well with only minor deviations. Citrate was adsorbed by anion exchange resins, resulting in a higher Ca^2+^ concentration downstream of the adsorbent cartridges during the first hour of treatment.

**Conclusions:**

The anion exchange resin depletes citrate, leading to an increased Ca^2+^ concentration in the extracorporeal circuit, which may cause an increased risk of clotting during the first hour of treatment. An increase of citrate infusion during the first hour of treatment should therefore be considered to compensate for the adsorption of citrate.

## Introduction

In patients suffering from liver disease with impaired coagulation, heparin can further contribute to hemorrhagic complications during extracorporeal liver support therapy,^1^ as it exerts its anticoagulatory activity not only in the extracorporeal circuit but also systemically. In regional citrate anticoagulation (RCA), the inhibition of the coagulation cascade as well as platelet activation are limited to the extracorporeal circuit, where the Ca^2+^ concentration is reduced by chelation with citrate. Before the blood is re-infused into the patient, the Ca^2+^ level is restored by an infusion of Ca^2+^. Furthermore, metabolization of citrate by the patient during and after the treatment leads to release of Ca^2+^ and contributes to the restoration of physiological Ca^2+^ level. Beyond anticoagulation, citrate exerts anti-inflammatory properties in extracorporeal blood purification^2–5^ and is associated with longer filter lifetimes.^6,7^

Therefore, the European Renal Association, the British Renal Association, as well as the KDIGO (Kidney Disease Improving Global Outcome) Working Group have recommended RCA for patients with increased bleeding risk,^8–10^ the KDIGO Working Group even for patients with low risk of bleeding. Meanwhile, RCA is widely applied also in extracorporeal liver support systems.^11–15^ Several authors have reported clotting events during adsorbent-based liver support therapy.^12,15,16^ In a recent study, Prometheus treatments of patients suffering from alcoholic liver disease had to be occasionally terminated prematurely due to filter clotting.^15^ Filter clotting was also reported during MARS treatment at a citrate concentration in blood of 3.1 mmol/L, where Ca^2+^ post filter was maintained at 0.22–0.44 mmol/L.^12^ Besides the disturbed coagulation in liver failure, interactions between adsorbents and anticoagulants in the extracoporeal circuit should be considered to contribute to these complications.

Both, in the Prometheus and the MARS liver support systems, anion exchange resins are used to eliminate negatively charged unconjugated bilirubin. However, in addition to the adsorption of their target molecules, clotting factors II and X as well as protein C were shown to be depleted by anion exchange resins in vivo and in vitro.^17^ The adsorption of these negatively charged molecules to the anion exchange resins may induce further imbalances of the clotting system. Since citrate is negatively charged, anion exchangers should be considered to deplete this anticoagulant. Therefore, we aimed to assess the depletion of citrate in extracorporeal circuits of liver support.

## Materials and methods

All experiments were performed in accordance with the Declaration of Helsinki 2013. The study was approved by the Institutional Review Board of Danube University Krems.

### In vitro blood purification experiment setup

**Figure 1.**
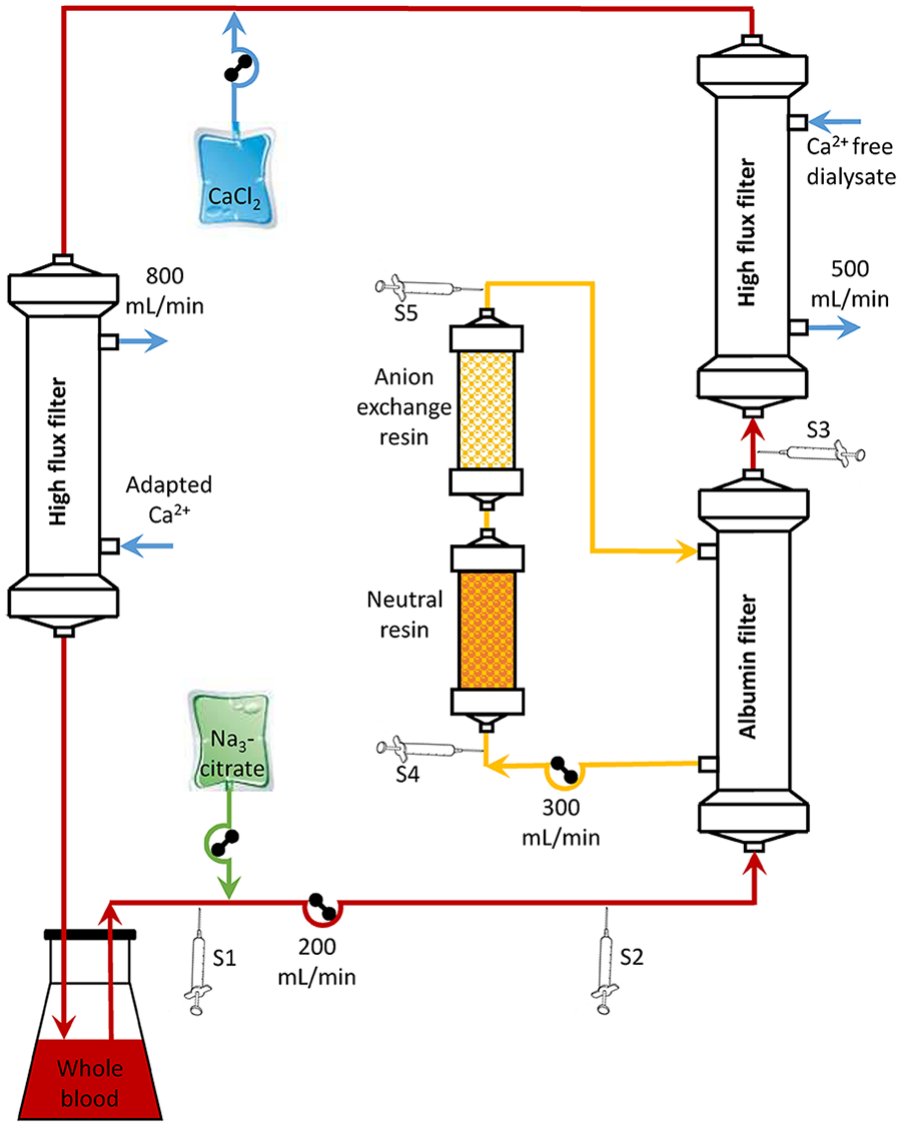
In vitro experimental set up. The blood circuit consisted of 1.2 L human whole blood (blood flow: Q_b_ = 200 mL/min), an albumin filter, a high-flux dialysis filter and a second high-flux dialysis filter to avoid citrate accumulation and to simulate different patients’ Ca^2+^ by using a dialysis fluid with different (0.9–1.5 mmol/L) Ca^2+^ concentrations. The filtrate circuit consisted of neutral and anion exchange resin cartridges (filtrate flow: Q_f_ = 300 mL/min). Citrate was infused according to blood flow, patient Ca^2+^, as well as targeted Ca^2+^ in the extracorporeal circuit. S1–S5: sampling positions: S1 pre citrate infusion, S2 pre albumin filter, S3 post albumin filter, S4 pre adsorbents, S5 post adsorbents.

To test the influence of adsorbents on the adsorption of citrate in extracorporeal circuits, in vitro experiments were performed (Figure 1; *n* = 3) with 1.2 L heparinized (9 IU/mL) human whole blood (Österreichisches Rotes Kreuz, Austria), withdrawn 1 day prior to the experiments.

For the experiments, the Prometheus Fresenius 4008H with an albumin permeable high cut-off filter (Albuflow, Fresenius Medical Care, Bad Homburg, Germany) and a high-flux dialysis filter (FX 60, Fresenius Medical Care) with calcium-free dialysis solution (SKF219/0, Fresenius Medical Care) were used. In the filtrate circuit, two columns containing a neutral resin (Prometh01, Fresenius Medical Care) and an anion exchange resin (Prometh02, Fresenius Medical Care) were incorporated. The blood flow rate was 200 mL/min, dialysis fluid flow 500 mL/min, and filtrate flow 300 mL/min.

To avoid citrate accumulation and to maintain a defined Ca^2+^ concentration in the blood pool, an additional high-flux dialysis filter (FX 60, Fresenius Medical Care) was integrated in the venous line after calcium substitution with a dialysis fluid flow rate of 800 mL/min to allow the maximum possible exchange rate. Dialysis fluid calcium was adjusted by mixing calcium-free (SKF219/0, Fresenius Medical Care) with calcium containing (SKF 213/4, Fresenius Medical Care) dialysis fluid to reach concentrations of 0.9, 1.2, or 1.5 mmol/L in order to simulate different Ca^2+^ plasma levels.

For RCA, 0.5 M tri-sodium citrate solution and 0.5 M CaCl_2_ solution (both from Provobispharm, Salzburg, Austria) were infused using an algorithm to adjust Ca^2+^ pre albumin filter to 0.2 or 0.3 mmol/L and to substitute calcium loss via the dialysis filter.^18^

Samples were drawn from the blood circuit pre citrate infusion as well as pre and post albumin filter, and from the filtrate circuit pre and post adsorbent cartridges at the beginning as well as after 0.5, 1, 2, 3, 4, 5, 6, 7, and 8 turn-overs of the pool volume. All samples were analyzed for citrate and Ca^2+^ (*n* = 3).

### Determination of the citrate adsorption capacity

To quantify and differentiate adsorption characteristics, adsorption isotherms of each adsorbent were determined in batch experiments with different quantities (0–10 mL) of adsorbents in 10 mL fresh frozen plasma with a citrate concentration of about 20 mmol/L (Österreichisches Rotes Kreuz, Austria). Citrate concentration at steady state was measured in supernatant after incubation for 6 h (*n* = 6).

### Measurements

Ca^2+^ was analyzed by an ion selective electrode (NOVA CRT 8, Nova Biomedical Waltham, USA). Citrate was quantified by a colorimetric test (R-Biopharm AG, Darmstadt, Germany) on an automated analyzer (Hitachi 902, Roche, Austria).

### Data analysis

For the blood purification experiments, only data with a valid citrate dosage were included. Data were excluded if the difference between the intended and the measured patient Ca^2+^ was > 0.1 mmol/L. Statistical analysis was conducted using SPSS 19 (SPSS Inc., Chicago, USA).

For adsorption isotherm determination, the following formulas were used:

The distribution volume of citrate consists of the plasma volume, the volume of pores plus interadsorbent volume and differs depending on adsorbent volume used.

$$V_{d}=V_{pl}+V_{po}$$

$V_{d}$ *. . . . . . . . . citrate distribution volume [mL]*

$V_{pl}$ *. . . . . . . . . plasma volume [mL]*

$V_{po}$ *. . . . . . . . . pore and interadsorbent volume [mL]*

Pore and interadsorbent volume was estimated by mass difference of dry versus wet adsorbent of each sample. Initial citrate concentration was calculated from plasma citrate concentration measured without adsorbent by considering the different distribution volumes due to different adsorbent volumes.

$$Ci_{0}=Ci_{pl_{0}}*(V_{pl}/V_{d})$$

$Ci_{0}$ *. . . . . . . initial citrate concentration [mmol/L]*

$Ci_{pl_{0}}$ *. . . . . . initial citrate concentration without adsorbent [mmol/L]*

Adsorbed citrate was calculated by the difference of citrate concentrations in supernatant at steady state and initial citrate concentration proportional to distribution volume.

$$Ci_{ads}=(Ci_{0}-Ci_{ss})*V_{d}$$

$Ci_{ads}$ *. . . . . adsorbed citrate [µmol]*

$Ci_{ss}$. . . . . . . *citrate concentration of supernatant at steady state [mmol/L]*

Adsorption capacity was calculated as adsorbed citrate concentration at equilibrium per g of dry adsorbent.

$$q_{e}=Ci_{ads}/m_{ads}$$

$q_{e}$ *. . . . . . . citrate adsorbed at equilibrium per g of adsorbent [µmol/g]*

$m_{ads}$ *. . . . . . dry mass of adsorbent [g]*

To be able to extrapolate to infinite, the reciprocal of adsorbed citrate per g of adsorbent and citrate concentration at steady state were used in adsorption isotherm. The reciprocal of adsorption capacity was determined by the resulting linear function *f(x)* at *x = 0*.

## Results

### In vitro blood purification experiments

Overall 148 out of 166 samples of the blood circuit and 82 out of 88 samples of the filtrate circuit were included in data analysis. Ca^2+^ concentrations pre and post albumin filter and pre and post adsorbents are summarized in Table 1.

**Table 1.** Ca^2+^ at different sample positions and in different anticoagulation groups.

Anticoa-gulation group	Ca^2+^ Pre albumin filter [mmol/L]			Ca^2+^ Post albumin filter [mmol/L]			Ca^2+^ Pre adsorbent [mmol/L]			Ca^2+^ Post adsorbent [mmol/L]		
	*n*	mean	±SD	*n*	mean	±SD	*n*	mean	±SD	*n*	mean	±SD
Total	148	0.24	±0.06	148	0.36	±0.11	82	0.30	±0.11	82	0.37	±0.17
0.2 mmol/L	75	0.19	±0.02	75	0.32	±0.10	42	0.27	±0.12	42	0.34	±0.19
0.3 mmol/L	73	0.29	±0.03	73	0.41	±0.09	40	0.34	±0.10	40	0.40	±0.14

**Figure 2.**
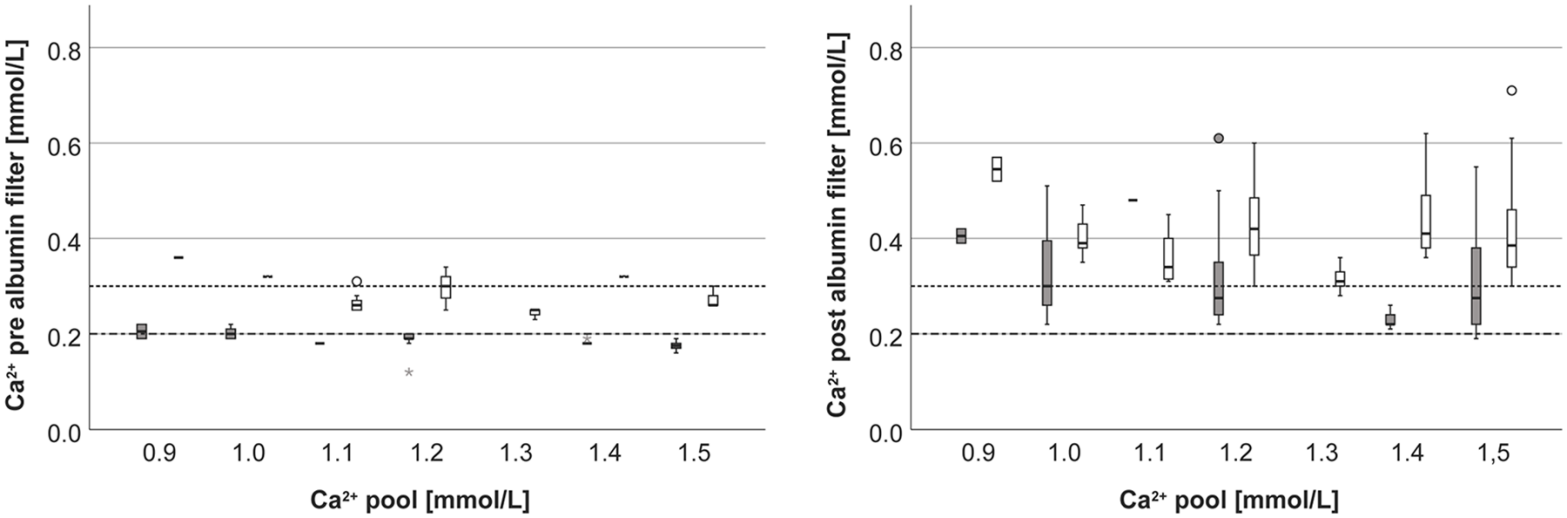
Ca^2+^ pre albumin filter (left), post albumin filter (right) at different pool Ca^2+^ concentrations (gray: anticoagulation group 0.2 mmol/L, white: anticoagulation group 0.3 mmol/L).

As shown in Figure 2, the target Ca^2+^ pre albumin filter could be reached well, whereas post albumin filter Ca^2+^ were above the target concentrations.

**Figure 3.**
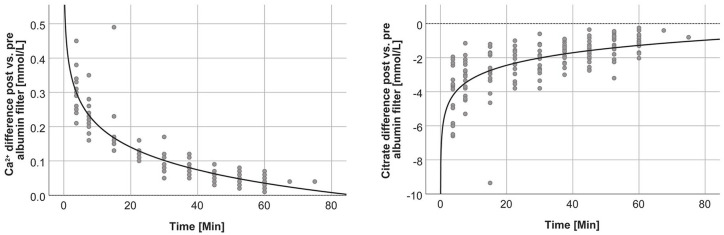
Difference between post and pre albumin filter Ca^2+^ and citrate concentrations over time (*y* = 0.425–0.095log(x), *r*² = 0.818 and *y* = –5.632+1.064log(x), *r*² = 0.446, respectively).

The difference between post and pre albumin filter Ca^2+^ was reduced over time (Figure 3). These findings are consistent with citrate concentrations which are lower post versus pre albumin filter and that their difference is also reduced over time.

**Figure 4.**
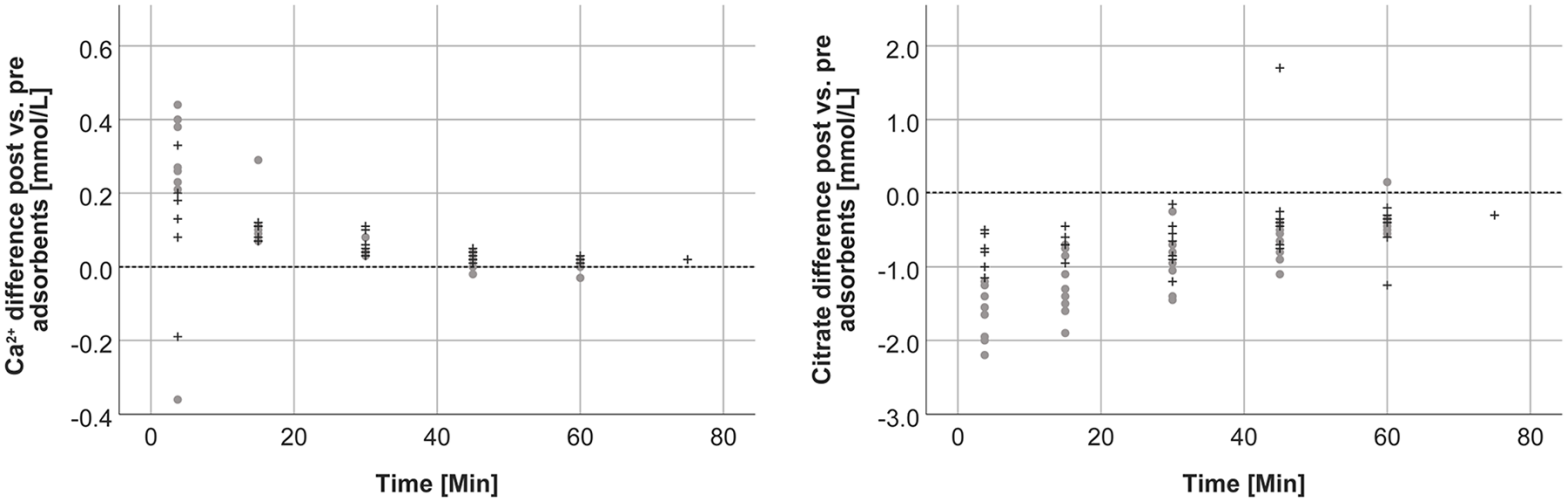
Difference between post and pre adsorbent Ca^2+^ and citrate concentrations over time (gray dots: anticoagulation group 0.2 mmol/L, black crosses: anticoagulation group 0.3 mmol/L).

In accordance with the findings in the blood circuit, we observed a reduction of citrate and an increase of Ca^2+^ in the filtrate circuit between pre and post adsorbent concentrations (Figure 4).

As shown in Table 2, about one-third of all post albumin filter Ca^2+^ samples exceeded a Ca^2+^ of 0.4 mmol/L (Ca^2+^: 0.49 ± 0.07 mmol/L), 22.7% in the anticoagulation group 0.2 mmol/L (Ca^2+^: 0.48 ± 0,05 mmol/L), and 45.2% (0.49 ± 0.07 mmol/L) in 0.3 mmol/L anticoagulation group. After the first 15 min the ratio of Ca^2+^ >0.4 mmol/L is reduced to 17.2% (Ca^2+^: 0.45 ± 0.05 mmol/L), to 1.8% (*n* = 1) in anticoagulation group 0.2 mmol/L (Ca^2+^: 0.61 mmol/L) but still to 32.2% in anticoagulation group 0.3 mmol/L (Ca^2+^: 0.44 ± 0.03 mmol/L).

**Table 2.** Percentage of measured Ca^2+^ exceeding the target concentrations 0.4 and 0.35 mmol/L post albumin filter from 0 to 60 min and from 15 to 60 min.

Anticoagulation group	% of measured Ca^2+^ samples exceeding target Ca^2+^			
	0–60 min		15–60 min	
	>0.4 mmol/L	>0.35 mmol/L	>0.4 mmol/L	>0.35 mmol/L
Total	33.8	50.0	17.2	36.2
0.2 mmol/L	22.7	29.3	1.8	7.0
0.3 mmol/L	45.2	71.2	32.2	64.4

### Adsorption capacity determination

**Figure 5.**
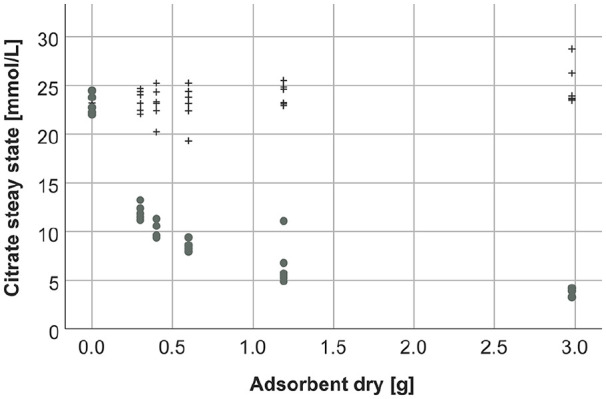
Citrate concentrations in the supernatant at steady state depending on different quantities of adsorbents (black crosses: neutral resin, gray dots: anion exchange resin).

For neutral resin, citrate concentrations in the supernatant corrected by the distribution volume remained high with increasing adsorbent quantities whereas a steep decline of citrate concentrations was observed for the anion exchange resin (Figure 5).

**Figure 6.**
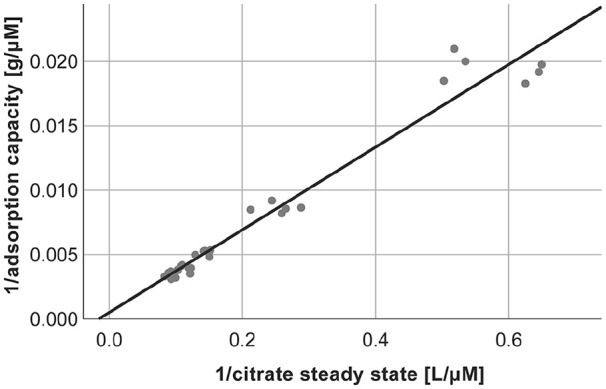
Adsorption isotherm of citrate for anion exchange resin (*y* = 0.001 + 0.032x, *r*² = 0.961).

The citrate adsorption capacity for anion exchange resin calculated by the adsorption isotherm (Figure 6) was 1.97 mmol/g dry adsorbent. Since the neutral resin did not adsorb citrate, there is no correlation, and the adsorption capacity for the neutral resin could not be calculated.

## Discussion

Clotting problems during adsorbent-based extracorporeal liver support can be due to disturbances of the clotting system in patients suffering from liver diseases. Moreover, adsorption of negatively charged clotting factors and its regulators by anion exchange resins can aggravate the imbalance of the coagulation system, favoring its activation.^17^ Although RCA is increasingly applied in extracorporeal liver support, the extent of citrate adsorption to anion exchange resins has not been evaluated systematically to date.

In this study, in vitro blood purification set ups were used to assess the anticoagulation in the extracorporeal circuit. Pre albumin filter target Ca^2+^ concentrations were achieved well with only minor deviations, while citrate adsorption by the anion exchange cartridges resulted in a higher Ca^2+^ post albumin filter. Post albumin filter, a dialysis filter is incorporated into the system to eliminate water-soluble toxins. Insufficient anticoagulation at the dialyzer entrance may be detrimental. At the filter entrance, blood cells are exposed to increased shear stress and flow disturbances, which force activation of platelets and raise the risk of coagulation activation. To inhibit the activation of coagulation, a reduction of Ca^2+^ to ⩽0.4 mmol/L is widely applied in RCA.^19^ In our study, for more than one-third of all analyzed samples, the post albumin filter anticoagulation status was not satisfactory. Especially when targeting a Ca^2+^ concentration of 0.3 mmol/L in the extracorporeal circuit, a clinically relevant target concentration,^20^ almost one-half of the measured samples exceeded a Ca^2+^ concentration of 0.4 mmol/L. For the 0.2 mmol/L anticoagulation group – a concentration only used by few dialysis centers^21^ – still about 23% of all samples showed an increased risk of clotting.

Adsorption of citrate almost reached steady state after 1 h of treatment, since after 1 h the difference between Ca^2+^ concentrations pre and post albumin filter was negligible. The risk of clotting based on elevated Ca^2+^ concentrations thus mainly exists during the first hour of treatment.

To quantify the adsorption of citrate, we calculated the adsorption capacity based on adsorption isotherms. We observed a strong reduction of citrate by the anion exchange resin, whereas the slight decrease of citrate, which was detected for the neutral resin, was reflecting dilution by different quantities of adsorbent. Theoretically, one column of anion exchange resin can bind up to 240 mmol citrate. While this calculated adsorption capacity is a theoretical value, it clearly shows a strong adsorption of citrate by the anion exchange resin. To reduce the risk of clotting during the first hour of treatment, an increase of the citrate infusion rate in order to compensate for citrate adsorption could be considered. Therefore, a pre dialyzer Ca^2+^ concentration ≤0.4 mmol/L should be targeted to assure an adequate anticoagulation status in the whole extracorporeal system. Another option would be to equilibrate the adsorbent with citrate solution before the treatment. However, this would require further studies as well as a risk assessment. Unpublished data from our group show that pretreatment of the adsorbent with citrate does not significantly affect its adsorption capacity for toxins such as bilirubin.

In contrast to the anion exchange resin, the neutral resin adsorbent did not adsorb citrate. Therefore, adsorption of citrate by neutral resins, which are applied for cytokine adsorption in whole blood in sepsis and other severe inflammatory diseases, such as the current COVID-19, seems also unlikely.

In conclusion, we found that the anion exchange resin adsorbed citrate, resulting in an increased Ca^2+^ concentration in the extracorporeal circuit and an increased risk of clotting during the first hour of treatment.
